# First-line managers dealing with different management approaches

**DOI:** 10.1108/LHS-09-2018-0046

**Published:** 2019-09-26

**Authors:** Annika Strömberg, Maria Engström, Heidi Hagerman, Bernice Skytt

**Affiliations:** 1 Faculty of Health and Occupational Studies, Department of Health and Caring Sciences University of Gävle, Gävle, Sweden; 2 Faculty of Health and Occupational Studies, Department of Health and Caring Sciences University of Gävle, Gävle, Sweden; Department of Public Health and Caring Sciences, Uppsala University, Uppsala, Sweden and Nursing Department, Medicine and Health College, Lishui University, Lishui, China; 3 Faculty of Health and Occupational Studies, Department of Health and Caring Sciences University of Gävle, Gävle, Sweden and Department of Public Health and Caring Sciences, Uppsala University, Uppsala, Sweden

**Keywords:** Leadership, Elderly care, Value-orientation, First-line manager, Production-orientation

## Abstract

**Purpose:**

The purpose of this paper is to contribute new knowledge about how first line managers (FLMs) in elderly care perceive their situation, with a focus on differences in management approaches at the intersection of the central and local parts of the organization.

**Design/methodology/approach:**

The present study has a qualitative approach and is part of a larger project on FLMs in elderly care. The results presented here are based on a secondary analysis of 15 of the total of 28 interviews carried out in the project.

**Findings:**

The main results are twofold: the majority of FLMs perceived differences in management approaches between local and central management; the differences caused some struggle because FLMs perceived that the management system did not support the differences. The two main aspects that caused the FLMs to struggle were differences in the foci of the management levels and difficulties in influencing the conditions of management.

**Originality/value:**

The results contribute to the debate on what aspects are important to sustainable management of elderly care. It is common knowledge that FLMs have a complex position, intermediate to the central, upper level management and their subordinates at the local level – levels with different foci and interests. The study contributes new knowledge about what these differences consist of and the dilemmas they cause and offers suggestions as to what can be done to reduce both energy waste and the risk of low job satisfaction.

## Introduction

The situation for first line managers (FLMs) in municipal elderly care is often described as complex and challenging (Ekholm, 2012; Hagerman *et al.*, 2015). The role of FLMs involves dealing with demands and expectations that come from several directions as well as balancing a variety of interests (cf. Björk, 2013). The FLMs often work close to daily operations at their units and have responsibility for services, staff and finances. Their position is intermediate to the central, upper level management and their subordinates at the local level (Lutz and Olsson, 2011). The present study aims to contribute new knowledge about how FLMs perceive their own local management and central management. By central management, we mean the hierarchy “above” the FLMs, i.e. levels of middle managers and central management. By local management, we mean the management the FLMs are responsible for providing in their respective organizations.

Elderly care in Sweden is experiencing great challenges, with high rates of sick leave and difficulties in recruiting personal (Ministry of Health and Social Affairs, 2018). The leadership executed by managers has been shown to be an important aspect of a healthy work environment, with regard to employees’ job satisfaction (Decker *et al.*, 2009; Westerberg and Tafvelin, 2014; van der Borg *et al.*, 2017), job turnover (Brannon *et al.*, 2002), perception of empowerment (Hagerman *et al.*, 2017), health (Wreder *et al.*, 2008; Ljungblad *et al.*, 2014) as well as quality of care in the health sector (Westerberg and Tafvelin, 2014). In that sense, in the context of elderly care, FLMs are central to ensuring a healthy work environment and reducing the high rates of sick leave and turnover.

Researchers have argued that management of the health-care sector is complicated because managers are supposed to simultaneously deal with the needs and expectations of different stakeholders, such as the employees, patients, relatives, different professionals and central management (Ekholm, 2012; Hasenfeld, 2015; Peters, 2018). These needs and expectations sometimes are contradictory and difficult to combine. Glouberman and Mintzberg (2001) describe these stakeholders as belonging to different “worlds,” with different sets of activities, ways of organizing and mindsets. Glouberman and Mintzberg (2001) point out, for example, the different logics of the world of care and the world of control. Kristiansen *et al.* (2015) discuss how the contradictions between the managerial logic of healthcare – characterized by increased efficiency demands with a strict focus on budgetary discipline and financial control – and the traditional professional logic – focused on ethics and quality of care – have affected the organization of daily work for nurses. Through continuous sense-making, nurses have coped with and handled the contradicting logics, which has triggered constant reorientation in their organization of daily work. The situation described above puts managers in elderly care in a central but also complex position, especially when there are several levels of management. It is reasonable to assume that there might be contradicting interests between management levels and that, in this complex situation, there is also room for more than one management approach (Ekholm, 2012). Results from a study of FLMs in Sweden and Egypt (Abdelrazek *et al.*, 2010) revealed higher values for achievement orientation, organizational view and political savvy, job satisfaction, structural and psychological empowerment in Egypt compared to Sweden. The authors discuss the managerial levels as one of several possible explanations for these findings, in that many of the Egyptian FLMs were highest up in the organizations (Abdelrazek *et al.*, 2010).

### Theoretical framework – different management approaches

The concepts of management and leadership can be seen as overlapping, where management often is related to administration, planning and decision-making, while leadership is more about influencing others and processing change (Bush, 2008). Management and leadership are described as two processes: management seeks to produce predictability and order, while leadership produces movement and change. Either one or several persons in an organization can have the formal responsibility for management and leadership processes (Yukl, 2013). Leaders can also be both formal and informal, but in the present article, we focus on the formal FLMs’ role and management of elderly care.

Different ways of leading involve different approaches to providing direction, implementing plans and motivating people. That means performance of managerial duties is guided by the management approach (Yukl, 2013). Different management approaches focus on disparate issues and therefore have various needs for tools and support systems, here called management systems. Lindvall (2001) argues that management systems need to be set up differently depending on the management approach taken. Lindvall (2001) suggests that market-driven operations are primarily supposed to meet market demands and adapt organizational and financial management to market expectations and requirements. This entails a business-like approach to management (Lindvall, 2001). In the case of operations that are not subject to competition, however, production itself can be more in focus, starting from management’s own preferences concerning organizational development (Drucker, 1999). In this respect, non-competition conditions can take another approach to management, thus using another management system. Lindvall (2001) refers to these two different management approaches as production-oriented and value-oriented management. Research on leadership is often focused on different leadership styles and how these styles affect organizations in various ways. In the present study, the focus is not on leadership styles but on the formal management role. We have therefore chosen to take our point of departure in Lindvall’s (2001) model of management approaches.

The two approaches presented by Lindvall (2001) can be seen as dichotomies or extremes; they are illustrated by keywords in Table I. The dichotomies can serve as a theoretical tool for sorting management approaches, though in practice management approaches probably do not belong to one or the other extreme, but fall somewhere in between. Nonetheless, using these dichotomies can contribute to the discussion about FLMs’ role, which is often described as complex and demanding.

Lindvall (2001) explains that the production-oriented management approach assumes a context with stable markets and relatively little competition, where financial control instruments such as budgeting and cost accounting constitute satisfactory bases for decision-making (Johnson and Kaplan, 1987). This approach of management duty involves supervising individuals and co-ordinating their activities in an efficient manner (Merchant and Van der Stede, 2007).

The value-orientated management approach has developed as a consequence of changes in the context surrounding organizations (Lindvall, 2001). Increased competition and tightening economic frameworks have brought about a need for flexibility and opportunities for rapid adaptation. In addition, there has been a change in the outlook on employees and their role in the organization, implying that competence may constitute the most important resource (Johnson and Kaplan, 1987). Moreover, within health care, there in an emphasis on enabling staff empowerment, shared decision-making (McCormack and McCance, 2010) as well as empowering structures (Spence Laschinger *et al.*, 2010). The value-orientated management approach emphasizes the value of the workplace culture as an instrument for management, meaning that organizational norms and values must be taken into consideration (Merchant and Van der Stede, 2007).

The aim of the present article is to contribute to the knowledge about how FLMs in elderly care perceive their situation, with a focus on differences in management approaches at the intersection of central and local parts of the organization.

## Method

The present study has a qualitative approach using deductive content analysis (Patton, 2002) and is part of a larger project looking at FLMs in elderly care (Hagerman *et al.*, 2015; Skytt *et al.*, 2015; Hagerman *et al.*, 2018). The aim of the larger project was to describe the situation for FLMs working in elderly care. The results presented here are based on a secondary analysis of 15 of the total of 28 interviews carried out in the project. By secondary analysis, we mean that the interviews have previously been analyzed for another study with a different aim (Long-Sutehall *et al.*, 2010).

### Sample and setting

Fifteen FLMs (7 women; 8 men) were included in the analysis. The number of interviews required was determined by the principle of data saturation. The 15 participants were chosen from the total of 28 to achieve variation in sex, age, experience as managers, educational background and the number of subordinates in the group of staff. The participants were employed in municipal (*n *=* *12) or private (*n *=* *3) elderly care facilities in Sweden. Their age ranged from 33 to 65 years. They had 0.5 to 30 years of experience as managers, most having a background in social work or nursing. Their leadership training varied from vocational to university level education. The average size of the groups of staff was 45 individuals, though this figure varied between 15 and 120. The inclusion criterion for the larger project was that participants should have occupied their current positions for more than six months.

### Data collection

For the larger project, semi-structured interviews were conducted from April 2011 to June 2012. The opening question was “Can you describe what you believe your work as a first-line manager comprises?” The subsequent questions focused on describing what the managerial role included, and how the interviewees experienced their working situation and their structural conditions. Probes such as “please tell me more” or “what does that mean for you” were used to clarify the working experience. The interviews were carried out at the FLMs' workplace or at the workplace of one of the authors; they were recorded on MP-3 players. The interviews lasted for 1.25-2.5 h.

In this secondary analysis focused on management approaches, some of the questions from the larger project were of special interest. Questions that caused the FLMs to describe their management approach and conditions related to management approaches were for example: “Can you describe what is included in your role as an FLM?”, “Can you describe your financial responsibility?”, “What does the responsibility for your employees include?” and “Can you describe the support and information you get from central management?” The supplementary questions, such as “what does that mean to you,” enabled the respondents to explain more about their management approach and how they handled the situation in relation to the descriptions of their role and the conditions around them.

### Data analysis

The present data analysis included 15 interviews, but the process began with the first author reading all the 28 transcribed interviews to get a feeling for the whole. First the interviews with female FLMs were read and subsequently the interviews with male FLMs. The reading of the interviews gave increased knowledge about the content, and the first step provided a picture in which the FLMs described differences between what they wanted to do in their role or believed in and what the management system allowed them to and what was rewarded by central management. The picture was that they perceived different approaches on the part of central and local management.

Further analyzing and categorizing a theoretical framework describing the different management approaches were needed to help sort the different perceptions. We therefore used Lindvall’s (2001) model of two dichotomies or extremes of management approaches to sort the material. This step of the analysis began with interviews with the three most experienced female FLMs. The argument for doing so was the assumption that the more experience you have, the more likely it is that you have had time to reflect on your role, the management system and working conditions. After this step, the interviewees were chosen to achieve variation as described above. The interview transcripts were read in their entirety several times to get a sense of the whole. Thereafter, parts of the text that described issues related to management approaches were identified in relation to Lindvall’s description of the two dichotomies.

The parts of the transcriptions describing issues related to management were labeled with the keywords from Lindvall’s model, Table I (Lindvall, 2001). Texts from the transcribed interviews were labeled using the following keywords: control versus empowerment, reaction versus proaction, finances versus competence, and so on. After 12 interviews had been analyzed, all the keywords in Table I had been used as labels. That means all the keywords had been associated with content-related substance. After 15 interviews, we concluded that data saturation has been achieved (Polit and Beck, 2012).

**Figure 1.**
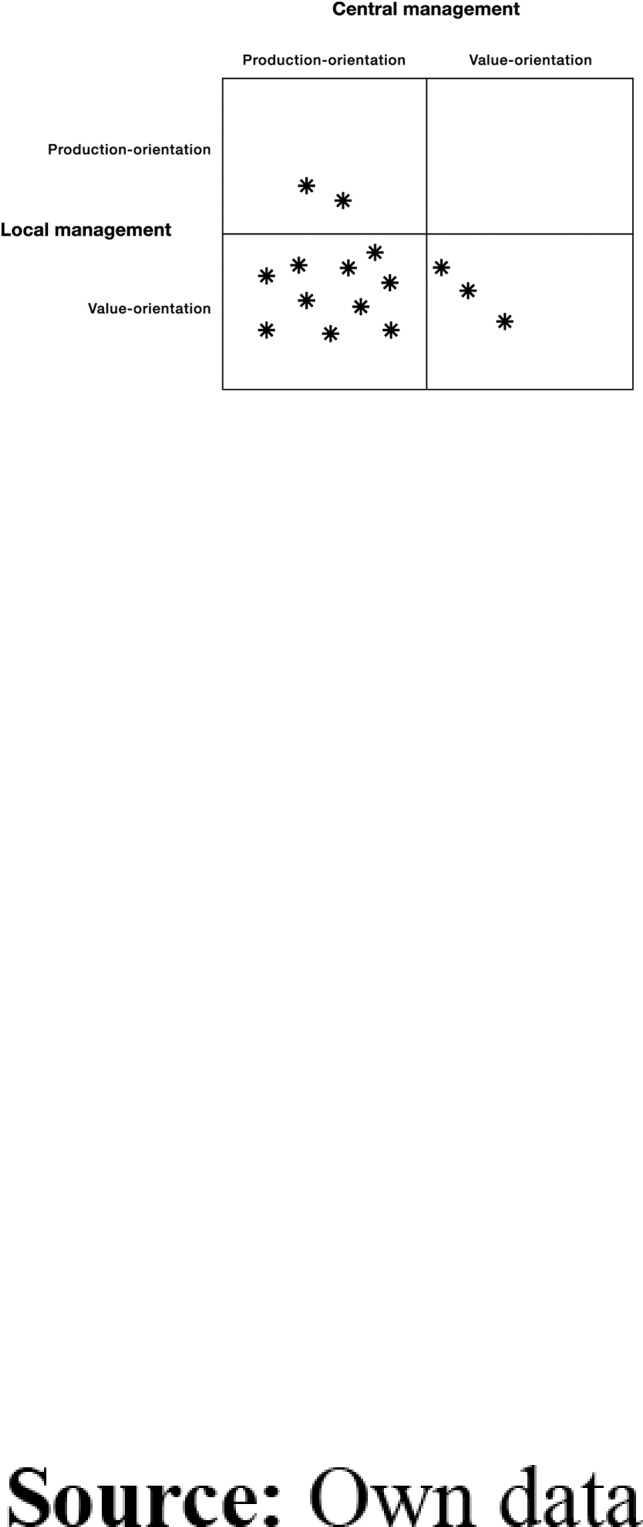
Patterns of informants’ views on local and central management approaches

Subsequently, the labels applied in the 15 interviews were categorized as descriptions of either local management or central management. Summarizing the codes from each interview gave an overall picture of the FLMs’ perceptions of local and central management as either production- or value-oriented. The analysis resulted in descriptions of the meaning of each category (see Table II). Based on the perspectives production-orientation versus value-orientation and the perspectives local issues (governed by the FLM) versus central issues (governed by central management), a system of four categories was developed and the interviews were plotted on a 2 × 2 table (Figure 1).

To further analyze what it meant when an interview was categorized in one of quadrants, the interview transcripts were reread. The question was what implications the perceived situation had for the FLMs’ work situation. In this way, a theme was abstracted concerning the FLMs’ perceptions of the interplay between the local and central management approaches. The overarching theme was: *A struggle arises when differences in management approaches are perceived.* Each step of the analysis was discussed among the group of authors until consensus was reached.

### Ethical considerations

The study was approved by the Regional Ethical Review Board (reg. no. 2010/192). The FLMs received oral and written information about the study. Participation was strictly voluntary; they were informed that they could withdraw from the study at any time and assured confidentiality.

## Results

The results are presented in one theme and four categories (Table II). The categories concern how the FLMs described the central and local management approaches in relation to the two perspectives: production-orientation and value-orientation (cf. Table I about different approaches). The theme shows how the FLMs perceived the differences in approaches.

### A struggle arises when differences in management approaches are perceived

The overarching theme in the analysis was: *A struggle arises when differences in management approaches are perceived,* which articulates the ongoing strife the FLMs experienced when trying to deal with the perceived differences in local and central management approaches. The results show that this was reflected in the management system, which influenced whether and how FLMs, in relation to their own management approach, could act in their FLM role.

The analysis demonstrates that the informants had different perceptions of both central and their own local management and that, combining the approaches in a 2 × 2 table, individual FLMs fell into one of four quadrants indicating different patterns. The distribution of informants across these four quadrants is shown in Figure 1.

Figure 1 shows that the majority of interviewees described a production-oriented central management. At the same time, the FLMs’ portrayals of their own local management and leadership were characterized by value-orientation. The analysis demonstrates that, for the FLMs who found themselves in a situation with different management approaches, there were two distinct aspects that resulted in struggles when dealing with these differences:

#### Differences in the focus of management.

The FLMs described that central management focused on finances, while the most important resource for local management was the staff, their competence and the quality of care. The control systems were governed in financial terms and gave the FLMs poor information about the other parts of business administration:

Yes, economy is the governing force […] It’s quite tough, trying to fulfil the demands of keeping to the budget versus the care that has to be provided. […] Last autumn, I was over budget. Then I explained to the accountants that we have two alternatives; either I ensure that the residents receive the care that they are entitled to, which is my duty, or I keep in line with the resources we have been allocated and cancel their care, but then you will have to give the residents/clients the information that we are unable to provide them with the care they have been granted. (FLM 6)

When the budget is drafted you are supposed to plan it according to the requirements of the coming year. But that’s just a lot of guff […] and the budget is certainly not set according to the requirements either, we just get a pot of money. […] all you can do is draft a shadow budget […] and then you keep your fingers crossed. […] We have to find another model that is based on looking at care requirements […] The way it is today, you get the same amount of money for each resident […] But you still have to try to break those boundaries sometimes and stretch them a little. Both in terms of resources and in terms of your own mandate. Like for example when I hire additional staff without really having the financial means. (FLM 5)

#### Difficulties in influencing the conditions of management.

The FLMs described difficulties in influencing recruitment, costs and revenues. They also described problems associated with large staff groups, particularly being able to influence the norms and cultures within working groups:

What I find hardest in my role as a manager at the moment is the cases, the clients we get […] I’m in an area where the majority aren’t elderly people, the majority are younger people with mental health problems […] our organization isn’t designed to deal with that and it can be frustrating. We can’t do a good enough job for these people to get back on their feet again. We don’t have that capability […] and then we get a lot of crap when they don’t get back on their feet […]. (FLM 7)

It can sometimes be frustrating when you’re doing skills development work with the staff group, and then you can’t always influence who you’re going to get, so you might get someone who hasn’t worked well in another group so they’re transferred […] You don’t have any say in that, you just have to take whoever you’re given. (FLM 6)

In the following section, we describe the four categories concerning how the FLMs described the central and local management approaches in relation to the two perspectives: production-orientation and value-orientation.

### Production-oriented central management – control of finances

The FLMs described the organization’s economic framework as a major control factor. Rules and routines concerning financial control were clear and set by central management. The positive aspect of this clarity was that operations and their content were governed by explicit rules. The FLMs worked according to a given budget, the goal being that this budget should be kept. The formalized management systems for follow-up and feedback were thus focused on costs and budget goals:

Once I’ve exceeded that budget, the deed is done. You must work actively with the budget throughout the year. That’s why we’re having meetings with our boss. So we’re under quite a bit of pressure when it comes to finances […] (FLM 1).

The resource that affected costs the most was staff working hours. Given a fixed budget, piecing together and scheduling staff working hours were a central component of cost control. The tight budget frameworks meant that FLMs had to use manpower resources as efficiently as possible, such that direct care was prioritized rather than administration around it, for instance, time for meetings and planning.

The operations were allotted revenues according to a central classification of the residents’ respective care burdens. The units themselves could not influence which residents would be sent to them. Thus, revenues could not be influenced so as to create better financial conditions for the organization. In addition to the top-down management of costs and revenues, staff appointments were also centrally controlled. Staff were employed within the municipality, which meant that they, taking into account seniority rules, etc., were placed where they were most needed, rather than based on competence and work results:

I wish it were easier to get rid of staff and pick the right ones. But that doesn’t work because it's policy and rules that decide. (FLM 2)

Another expression of production orientation was that work toward change was carried out in the context of projects outside the regular activities and budget. Client assessments of care were conducted in the form of a Customer Satisfaction Index. However, it was not evident from the FLMs’ descriptions that the results of these measurements had any effect on allocation of funding. Thus, there was no link between client experiences and funding. Consequently, managers were not in a financial position to influence client experiences through, e.g. work toward change.

### Value-oriented central management – vision and holistic perspective

The FLMs did not talk quite as often about value-oriented central management. The approach was seen in some FLMs’ descriptions of management systems using scorecards with several focus areas that did not only include financial measures:

In the municipality, we have a so-called production plan. It governs our duties, our reports, everything. It deals with focus areas such as customers, finances, processes, co-workers. These areas involve all operations and make up our scorecard somehow (FLM 3).

However, it did not appear from the FLMs’ descriptions that these scorecards were used to achieve balanced management including all the perspectives observed. Rather, primarily one of the perspectives, the financial one, was followed up. One of the FLMs, however, portrayed all focus areas as governing, even centrally. The same person described the mission as strongly focused on development and change intended to meet clients’ needs:

We are developing as I said, our production manager, she is visionary. She has an image […] she’s been digging deep and gone over the whole ground. Simply to break new ground, it’s completely new (FLM 3).

In the interview, this person outlined great opportunities to shape the organization, leading and managing it based on an individual conviction concerning how it had to or should be run. Neither the economic framework nor the central rules were emphasized as being problematic. Rather, this person stressed the immediate superior’s constant anticipation of development and change, which was compatible with the person’s own outlook on management, where change was a natural part of daily operations. The same description of central management was provided by other participants in the study. To a great extent, their accounts concerned the existence of central management and systems for measurement and control that partly focused on following up costs, but also on aspects that could instead be associated with quality of care. In this way, operations could be regarded as characterized by tight control and follow-up, the norms determining which aspects were to be followed up.

### Production-oriented local management – cost efficiency

Production-oriented local management was rare among the FLMs descriptions. This approach was partly demonstrated by some of the FLMs, particularly those focusing on the need for local control, the reason being that central demands for cost-efficiency had to be met. One of the FLMs talked about the desire for a control system to ensure that staff members use their time in a resource-efficient manner:

The clock begins to run when you open the door with the key. Yes, that’s smart. Then you can’t get away, there’s no way to shirk your work. Yes, then it's automatic (FLM 4).

Another expression of a more production-oriented approach was that work was delegated because the FLMs felt a need to pass on tasks they themselves could not accomplish in time. In that situation, delegation can be considered a logical consequence of the workload rather than of belief in a leadership instrument to influence a working group with client-proximate responsibility, which is assumed by those who first see the need for specific measures. Several FLMs focused on their work as administrative heads and found little time to prioritize being a guiding-star and a coach in the midst of daily work:

I can say that I sit a lot at the computer, 75 per cent of my time I sit in front of the computer. (FLM 1)

The focus on cost-efficiency also influenced an approach marked by the position of heading a unit, maintaining the budget and reducing costs. This limited FLMs’ opportunities to be sensitive to staff members’ own wishes and to create room for addressing their needs in daily operations.

### Value-oriented local management – flourishing personnel

The majority of managers described their own local management as value oriented. This implies an approach where the client is in focus and where the most important resource in client encounters is the staff and their competence. The FLMs often described their function as involving an overall responsibility, where clients’ satisfaction, staff members’ well-being and financial balance were intertwined. They stated that, for them, finances were about more than merely figures and money. They declared that good finances could only be achieved if the staff found satisfaction in their work, which would be mirrored in clients’ experiences of care provision. They thought that if the staff feel good, then the clients will feel good too. In the long run, they thought this would also be reflected in the economic reports:

Because if you have happy and committed colleagues with a passion for their work, then you can cope with finances. Yes, then you’ll have satisfied clients and a sustainable situation (FLM 2).

This view meant that the work environment was an important focus area for the FLMs. To achieve this, FLMs endeavored to be involved in daily operations to the greatest extent possible. They felt that they needed to be close to the actual care provision to deal sensitively with demands that arose every day.

Several FLMs emphasized the value of a committed personnel group that assumed responsibility on their own. They stressed the importance of empowering staff with independent responsibility to increase commitment and, thus, the quality of care:

And of course the more responsibility they assume, the better it gets. They’ll reach their maximum capacity in this way […] It’s all a question of confidence and trust. There’s nothing more important than that. (FLM 5)

For me it was a psychosocial thing to incorporate the reticent ones, and I’ve seen them flourish, I’ve seen them grow. They have been given power, that’s really what they need. (FLM 3)

Many FLMs led organizations under constant change, both concerning external factors such as economic frameworks, rules, etc., and concerning changes in operations *per se*, including, e.g. residents’ health. To deal with these changes, the FLMs needed to run the organization so that it could be flexibly adapted to new conditions concerning, e.g. staffing needs. FLMs who had a value-oriented outlook stated that they had to act supportively, foresightedly and proactively, though without neglecting the financial side of the work. Financial planning and follow-up were important, but longer-range horizons were necessary and valuable as control instruments.

## Discussion

The main results of the present study are twofold: the majority of the FLMs perceived differences in management approaches between local and central management, and these differences caused some struggle because they perceived that the management system did not support the differences. The two main aspects that caused the FLMs to struggle were differences in the focus of management and difficulties influencing the conditions of management.

The present study further supports previous findings showing that FLMs in elderly care have to handle different perspectives, interests and needs (Glouberman and Mintzberg, 2001; Björk, 2013). When there is more than one management level, it is likely that the different levels will have different foci. This in itself creates an interface between different perspectives and interests. Therefore, the interesting point is not that there are differences, but how they are perceived, how they are handled and what their effects are. The goal of answering these questions is to create the preconditions for a healthy work environment in the long run.

The majority of the FLMs in the present study described central management as production-oriented, while portraying their own management as value-orientated. One explanation for why the majority of the FLMs interpreted central management as production orientated may be found in the high pressure placed on FLMs working in elderly care and the increased focus on efficiency. The FLMs had to handle these perceived differences in orientation, but doing so meant facing some struggles and dilemmas. This is in contrast to interviews with FLMs who perceived their own management approach to be equivalent to that of central management. The analysis of these interviews did not reveal the same kind of struggle in dealing with central management. In Figure 1, it is notable that none of the interviews fell into the quadrant for value-orientated central management and production-orientated local management. But perhaps this is not so remarkable. It seems more appropriate to place, for example, the label “control” on central management and more likely that local management will be characterized by, for example, “flexibility” in dealing with daily work, as opposed to the reverse attribution of characteristics.

The dilemmas and struggles between different management approaches expressed in the present study have similarities with the tension between different logics that Kristiansen *et al.* (2015) discuss. The authors demonstrate how, through continuous sense-making, nurses have coped with different logics that triggered constant reorientation in organization of their daily work. In the same way, Orevik *et al.* (2015) show how ward managers used different strategies to cope with conflicting demands for quality and efficiency. Although the FLMs in the present study handled the struggle, it can be seen as a waste of energy. Moreover, in the long run, if ways of coping and making sense are too difficult, and it seems impossible to maintain the professional logic of what is ethically right, the situation may cause moral distress and decreased job satisfaction (McCarthy and Deady, 2008; Hunt, 2014). Low job satisfaction is a common reason for nurses to leave their jobs, which in turn is thought to be related to, for example, negative effects on quality, efficiency and job security (Hayes *et al.*, 2006; O’Brian-Pallas *et al.*, 2010). Given the situation in elderly care, which is characterized by high levels of sick leave and turnover (Ministry of Health and Social Affairs, 2018), we wish to stress the importance of increasing knowledge about the struggle between different management approaches.

As demonstrated in the present results, there were two aspects that caused the FLMs to struggle. The different foci of the two management levels emerged in the described struggle between an emphasis on finances and the ambition to create a work situation in which the staff could see that work satisfaction was mirrored in clients’ experiences of care provision. The FLMs’ opinion seems to have been that the strongest voice was the managerial logic that focused on efficiency, which was visualized in budget monitoring. Even though the more professional logic did have a voice in discussions between different levels of management, the control system gave poor information about and was not affected by other aspects of business management (cf. Shanks *et al.*, 2014). The FLMs reported that the financial aspect was important, but that client satisfaction and staff wellbeing were prerequisites or crucial conditions for achieving a good financial outcome. Following that logic, the information and management system also need to take these aspects into consideration. Some of the FLMs in the study described routines for Customer Satisfaction Index reports, but also a lack of connection between report outcomes and financial matters. If other aspects of the organization such as these are not listened to and taken seriously, the result may be increased work stress for FLMs and a feeling of “voicelessness” (Orevik *et al.*, 2015; Gaudine and Beaton, 2002).

The FLMs pointed out that it was difficult for them to influence the conditions affecting costs, revenues and recruitment. At the same time, they had overall responsibility for the finances and were convinced that, to assume full responsibility, they needed to have tools that would enable them to influence and develop the teams and the culture at the workplace (Paliadelis, 2008). They also described a situation characterized by constant changes that made it crucial for them to be able to be flexible in how they managed the daily work. According to the FLMs, one interpretation of the situation is that they lack structural empowerment in their role. Studies have confirmed that structural empowerment is important and has a positive influence on patient outcomes, patient safety (Spencer and McLaren, 2016) and staff well-being (Hagerman *et al.*, 2016). The FLMs’ structural empowerment also has an impact on staff members’ ratings of their own structural empowerment and of their FLM’s leadership and management performance (Hagerman *et al.*, 2017). Kanter (1993) argues that four components work together to create structural empowerment: access to opportunities to learn and advance in one’s career, access to support from managers and subordinates, access to resources and access to the information needed to perform the work and assume responsibility. The FLMs described their limited possibilities to control resources and similarly limited opportunities to contribute to the budget process and strategic decision-making. They also reported lacking authority over the prerequisites for acquiring resources such as money and competence. In one respect, the FLMs had control over resources within the confines of the budget, but no influence on the prerequisites for, e.g. recruitment. Thus, they lacked structural empowerment with regard to resources (Kanter, 1993).

### Methodological considerations

Although the study for the larger project (within which this secondary analysis was made) was designed to acquire a broad picture of how managers perceived their work, attitudes related to management approaches emerged in the interviews. If different kinds of questions had been asked, the results might have been different. On the other hand, direct questions based on the problem areas presented here could have caused informants to respond more in line with their knowledge of what the situation should be like, i.e. to depict a more positive situation. The theoretical framework for the present study can be criticized as simple, describing different approaches as ideal types. The model is based on traditional business management ideas and was used as a tool to theoretically sort differences in management approaches. Given that purpose, it was useful to use a simple model that highlights differences in a clear way.

## Conclusions

The present results show that the FLMs struggle with the differences they experience in management approaches between the local and central levels. This struggle causes energy waste and in the long run may serve to increase turnover. The results show a struggle that involves two main aspects: differences in the focus of management and difficulties influencing the conditions of management. An important outcome of such struggling is a feeling of “voicelessness” when the FLMs try to deal with different interests and needs. The present findings indicate that it is the needs of the elderly and the staff working close to them that take the backseat to financial and managerial matters. That means that the various perspectives, interests and needs discussed do not exist on the same terms. Our main point is not that different management levels should use the same approach, but that the differences are natural and that it is important to deal with and make the most of them.

## Implications for practice

The present results contribute new knowledge about the complex situation for FLMs in elderly care and the struggle they face in dealing with differences in management approaches between central and local management. Through awareness of how the management approaches are perceived in practice and of what aspects are problematic, central management can promote management systems that give clearer signals as well as information systems that enable consistent and sustainable leadership and management. To take the different management approaches into account, the management system needs to deal with the demands and expectations of both central and local management. To accomplish this, there is a need for venues for dialog and a management system that supports different perspectives on the need for information, the proper level for decision-making and the mandate as concerns certain questions. The present results can serve as a starting point for dialogues about how the different approaches are realized in practice.

## Figures and Tables

**Table I.** The differences between production-orientation and value-orientation in management

Production-orientation	Value-orientation
Control	Empowerment
Reaction and details	Proaction and the whole
Finances and budget	Focus areas and competence
Change viewed as extra work	Change viewed as part of everyday work
Static	Flexible
System or norms	System and norms
Heading	Leading

**Source:** Based on Lindvall (2001)

**Table II.** Overview of theme and categories

Theme	Category
A struggle arises when differences in management approaches are perceived	Production-oriented central management – *Control of finances*
	Value-oriented central management – *Vision and holistic perspective*
	Production-oriented local management – *Cost efficiency*
	Value-oriented local management – *Flourishing personnel*

**Source:** Own data

## References

[ref001] Abdelrazek F., Skytt B., Aly M., El-Sabour M.A., Ibrahim N. and Engström M. (2010), “Leadership and management skills of first-line managers of elderly care and their work environment”, Journal of Nursing Management, Vol. 18 No. 6, pp. 736-745. 20840368 10.1111/j.1365-2834.2010.01132.x

[ref002] Björk L. (2013), “Contextualizing managerial work in local government organizations”, Dissertation, University of Gothenburg, Faculty of Social Sciences.

[ref003] Brannon D., Zinn J.S., Mor V. and Davis J. (2002), “An exploration of job, organizational, and environmental factors associated with high and low nursing assistant turnover”, The Gerontologist, Vol. 42 No. 2, pp. 159-168. 11914459 10.1093/geront/42.2.159

[ref004] Bush T. (2008), “From management to leadership – semantic or meaningful change?”, Educational Management Administration and Leadership, Vol. 36 No. 2, pp. 271-288.

[ref005] Decker F.H., Harris-Kojetin L.D. and Bercovitz A. (2009), “Intrinsic job satisfaction, overall satisfaction, and intention to leave the job among nursing assistants in nursing homes”, The Gerontologist, Vol. 49 No. 5, pp. 596-610. 19515636 10.1093/geront/gnp051

[ref006] Drucker P.F. (1999), Management Challenges for the 21st Century, Butterworth Heinemann, Oxford.

[ref007] Ekholm B. (2012), “Middle managers in elderly care under demands and expectations”, Leadership in Health Services, Vol. 25 No. 3, pp. 203-215.

[ref008] Gaudine A.P. and Beaton M.R. (2002), “Employed to go against one’s value: nurse managers’ accounts of ethical conflict with their organizations”, Canadian Journal of Nursing Research, Vol. 34 No. 2, pp. 7-34. 12424998

[ref009] Glouberman S. and Mintzberg H. (2001), “Managing the care of health and the cure of disease - part 1: differentiation”, Healthcare Management Review, Vol. 26 No. 1, pp. 56-69. 10.1097/00004010-200101000-00006 11233354

[ref010] Hagerman H., Engström M., Häggström E., Wadensten B. and Skytt B. (2015), “Male first-line managers’ experiences of the work situation in elderly care: an empowerment perspective”, Journal of Nursing Management, Vol. 23 No. 6, pp. 695-704. 24283766 10.1111/jonm.12197

[ref010a] Hagerman H., Engström M., Wadensten B. and Skytt B. (2018), “How do female first-line managers in elderly care experience their work situation? – An interview study”, Journal of Nursing Management, doi: 10.1111/jonm.12793. PMC7328729 31102540

[ref011] Hagerman H., Högberg H., Skytt B., Wadesten B. and Engström M. (2017), “Empowerment and performance of management of managers and subordinates in elderly care: a longitudinal and multilevel study”, Journal of Nursing Management, Vol. 25 No. 8, pp. 647-656. 28714218 10.1111/jonm.12504

[ref012] Hagerman H., Skytt B., Wadesten B., Högberg H. and Engström M. (2016), “A longitudinal study of working life among first-line managers in care of older adults”, Journal of Applied Nursing Research, Vol. 32, pp. 7-13. 27969055 10.1016/j.apnr.2016.03.003

[ref013] Hasenfeld Y. (2015), “What exactly is human services management? Human service organisations: management”, Leadership and Governance, Vol. 39 No. 1, pp. 1-5.

[ref014] Hayes L., O’Brian-Pallas L. and Duffield C. (2006), “Nursing turnover: a literature review”, International Journal of Nursing Studies, Vol. 43 No. 2, pp. 237-263. 15878771 10.1016/j.ijnurstu.2005.02.007

[ref015] Hunt D. (2014), “Does value congruence between nurses and supervisors effect job satisfaction and turnover?”, Journal of Nursing Management, Vol. 22 No. 5, pp. 572-582. 23829178 10.1111/jonm.12055

[ref016] Johnson T. and Kaplan R. (1987), Relevance Lost – The Rise and Fall of Management Accounting, Harvard Business School Press, Boston.

[ref017] Kanter R.M. (1993), Men and Women of the Corporation, Basic Books, New York, NY.

[ref018] Kristiansen M., Obstelder A. and Lotherington A.T. (2015), “Nurses’ sensemaking of contradicting logics: an underexplored aspect of organisational work in nursing homes”, Scandinavian Journal of Management, Vol. 31 No. 3, pp. 330-337.

[ref019] Lindvall J. (2001), Verksamhetsstyrning – Från Traditionell Ekonomistyrning till Modern Verksamhetsstyrning, Studentlitteratur, Lund.

[ref020] Ljungblad C., Granström F., Dellve L. and Åkerlind I. (2014), “Workplace health promotion and working conditions as determinants of employee health”, International Journal of Workplace Health Management, Vol. 7 No. 2, pp. 89-104.

[ref021] Long-Sutehall T., Sque M. and Addington-Hall j. (2010), “Secondary analysis of qualitative data: a valuable method for exploring sensitive issues with an elusive population?”, Journal of Research in Nursing, Vol. 16 No. 4, pp. 335-344.

[ref022] Lutz Ö. and Olsson C. (2011), “Employer perspectives in Swedish municipalities and county councils: facts and analyses for 2011”, Swedish Association of Local Authorities and Regions, Modintryckoffset, Stockholm.

[ref023] McCarthy J. and Deady R. (2008), “Moral distress reconsidered”, Nursing Ethics, Vol. 15 No. 2, pp. 254-262. 18272615 10.1177/0969733007086023

[ref024] McCormack B. and McCance T. (2010), Person-Centred Nursing: Theory and Practice, Wiley-Blackwell, London.

[ref025] Merchant K.A. and Van der Stede W.A. (2007), Management Control Systems, 2nd ed, Prentice Hall, Pearson Education Limited, Harlow, Essex.

[ref026] Ministry of Health and Social Affairs (2018), Framtidens Äldreomsorg - en Nationell Kvalitetsplan, Ministry of Health and Social Affairs, Stockholm.

[ref027] O’Brian-Pallas L., Murphy G., Shamian J., Li X. and Hayes L. (2010), “Impact and determinants of nurse turnover: a pan-Canadian study”, Journal of Nursing Management, Vol. 18 No. 8, pp. 1073-1086. 21073578 10.1111/j.1365-2834.2010.01167.x

[ref028] Orevik A., Vågen S.R., Axelsson S.B. and Axelsson R. (2015), “Quality, efficiency and integrity: value squeezes in management of hospital wards”, Journal of Nursing Management, Vol. 23, pp. 65-74. 23859046 10.1111/jonm.12084

[ref029] Paliadelis P.S. (2008), “The working world of nursing unit managers: responsibility without power”, Australian Health Review, Vol. 32 No. 2, pp. 256-264. 18447812 10.1071/ah080256

[ref030] Patton M.Q. (2002), Qualitative Research and Evaluation Methods, 3rd ed., Sage Publications, Thousand Oaks, CA.

[ref031] Peters S.C. (2018), “Defining social work leadership: a theoretical and conceptual review and analysis”, Journal of Social Work Practice, Vol. 32 No. 1, pp. 31-44.

[ref032] Polit D.F. and Beck C.T. (2012), Nursing Research: generating and Assessing Evidence for Nursing Practice, Wolters Kluwer Health/Lippincott Williams and Wilkins, Philadelphia, PA.

[ref033] Shanks E., Lundström T. and Wiklund S. (2014), “Middle managers in social work: professional identity and management in marketised welfare state”, British Journal of Social Work, Vol. 45 No. 6, pp. 1-17.

[ref038a] Skytt B., Hagerman H., Strömberg A. and Engström M. (2015), “First-line managers' descriptions and reflections regarding their staff's access to empowering structures”, Journal of Nursing Managemen, Vol. 23 No. 8, pp. 1003-1010. doi: 10.1111/jonm.12246. 25059511

[ref034] Spencer C. and McLaren S. (2016), “Empowerment in nurse leader groups in Middle management: a quantitative comparative investigation”, Journal of Clinical Nursing, Vol. 26 Nos 1/2, pp. 266-279. 10.1111/jocn.13426 27291299

[ref035] Spence Laschinger H.K., Gilbert S., Smith L.M. and Leslie K. (2010), “Towards a comprehensive theory of nurse/patient empowerment: applying Kanter’s empowerment theory to patient care”, Journal of Nursing Management, Vol. 18 No. 1, pp. 4-13. 20465724 10.1111/j.1365-2834.2009.01046.x

[ref036] van der Borg W.E., Verdonk P., Dauwerse L. and Abma T.A. (2017), ” “Work-related change in residential elderly care: trust, space and connectedness”, Human Relations, Vol. 70 No. 7, pp. 805-835. 28626242 10.1177/0018726716684199 PMC5464400

[ref037] Westerberg K. and Tafvelin S. (2014), “The importance of leadership style and psychosocial work environment to staff-assessed quality of care: implications for home help services”, Health &Amp; Social Care in the Community, Vol. 22 No. 5, pp. 461-468. 10.1111/hsc.12084 24313819

[ref038] Wreder Å., Gustavsson M. and Klefsjö B. (2008), “Management for sustainable health: a TQM-inspired model based on experiences taken from successful Swedish organizations”, International Journal of Quality and Reliability Management, Vol. 25 No. 6, pp. 561-584.

[ref039] Yukl G. (2013), Leadership in Organizations, 8th ed, Pearson, Essex.

